# UiO-66 Metal-Organic Framework Membranes: Structural Engineering for Separation Applications

**DOI:** 10.3390/membranes15010008

**Published:** 2025-01-01

**Authors:** Yanwei Sun

**Affiliations:** Faculty of Arts and Sciences, Beijing Normal University, Zhuhai 519087, China; sunyw@bnu.edu.cn

**Keywords:** MOF membrane, UiO-66, structure manipulation, synthesis methods, separation performance

## Abstract

Metal-organic frameworks (MOFs) have been recognized as promising materials for membrane-based separation technologies due to their exceptional porosity, structural tunability, and chemical stability. This review presents a comprehensive discussion of the advancements in structure engineering and design strategies that have been employed to optimize UiO-66 membranes for enhanced separation performance. Various synthesis methods for UiO-66 membranes are explored, with a focus on modulated approaches that incorporate different modulators to fine-tune nucleation rates and crystallization processes. The influence of preferred orientation, membrane thickness, pore size, pore surface chemistry, and hierarchical structures on the separation performance is concluded. By providing a consolidated overview of current research efforts and future directions in UiO-66 membrane development, this review aims to inspire further advancements in the field of separation technologies.

## 1. Introduction

Membrane technology presents considerable potential for improving the energy efficiency for separation process of molecules and ions, which currently contribute a significant share (10–15%) of global energy consumption [1,2,3]. When compared to traditional methods like distillation and adsorption, membrane technology provides various benefits, such as a more compact structure, reduced environmental impact and decreased energy usage. The successful industrial application of this technology, however, hinges on the development of high-performance membrane materials. Polymeric membranes are particularly attractive due to their solubility, low cost, and ease of processing. However, their performance is often limited by a trade-off between permeability and selectivity [4]. To overcome these limitations, researchers are increasingly focusing on membranes with ordered nanoporous structures, which offer improved stability, higher selectivity, and the potential to address the inherent trade-offs present in polymeric membranes.

Among these advanced materials, metal-organic frameworks (MOFs), or porous coordination polymers, have emerged as a novel class of highly crystalline porous materials. Characterized by coordination bonds between metal ions or clusters and organic ligands, MOFs are valued for their exceptional properties, including high Brunauer-Emmett-Teller (BET) surface area (often exceeding 10,000 m^2^/g), low density, tunable pore structures, high crystallinity, and versatile surface chemistry [5,6,7]. Zirconium(IV)-carboxylate metal-organic frameworks (Zr-MOFs) have recently attracted significant interest as potential membrane materials, primarily due to their remarkable stability [8].

**Figure 1:**
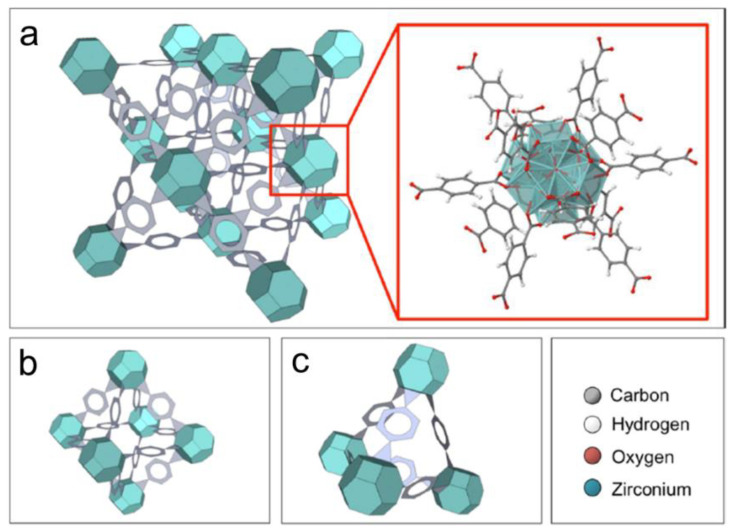
Illustration of the crystalline structure of UiO-66. (**a**) Structure of the inorganic building unit Zr_6_O_4_(OH)_4_; tetrahedral (**b**) and octahedral (**c**) cavities [10].

UiO-66, a pioneering Zr-MOF developed at the University of Oslo (UiO), initially synthesized using Zr clusters (Zr_6_O_4_(OH)_4_) as secondary building units (SBUs) and 1,4-benzenedicarboxylic acid (H_2_BDC) as linkers (Figure 1). The structure features octahedral (1.1 nm) and tetrahedral (0.8 nm) cavities, which are accessible through triangular windows measuring 0.6 nm [9]. UiO-66 allows for precise tuning of pore sizes through the selection of different organic linkers. This level of control is often greater than in many other MOFs, enabling optimized separation for specific gas mixtures.

The robust bonds between metal centers and organic ligands, driven by high framework connectivity, bond polarization, and high charge density, endow UiO-66 with outstanding chemical and thermal stability, which is thermally stable up to 500 °C [11]. While many other MOFs can suffer from degradation under high temperatures or reactive environments, UiO-66 maintains its structural integrity, allowing it to be used in a broader range of operational conditions. Additionally, compared to some MOFs, which inevitably undergo hydrolysis or structural collapse in moist conditions, UiO-66 is relatively stable, making it advantageous for processes involving humid gas mixtures [12].

Moreover, the hydroxylated Zr cluster in UiO-66 preferentially adsorb CO_2_, enabling effective separation of gas pairs such as CO_2_/N_2_ and CO_2_/CH_4_ [13,14]. Gas separations, especially CO_2_/N_2_ separation, are energy-intensive processes that contribute significantly to industrial energy consumption, underscoring the potential of UiO-66 membranes to mitigate both energy usage and carbon emissions. Additionally, the pore aperture of UiO-66 is larger than the kinetic diameter of water molecules but smaller than that of most salt ions, impurities (such as dyes), and organic solvents, making it advantageous for desalination [15,16]. By regulating ligand diversity, UiO-66 type MOFs also show potential for light hydrocarbon separation [17,18,19,20].

**Figure 2:**
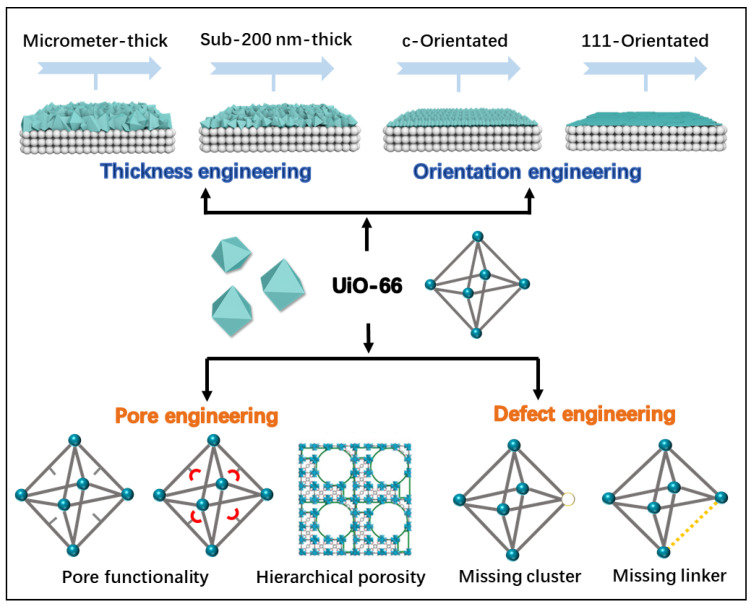
Manipulation strategies for improving separation performance on UiO-66 membranes.

To achieve high-efficiency molecular separation, precise structural manipulation of the UiO-66 membrane is essential. This involves optimizing pore size and distribution to enhance selectivity and tailoring the chemical environment within the pores to improve interactions with target molecules (Figure 2). For instance, modifying surface functional groups can fine-tune the affinity towards specific molecules, thereby enhancing their separation performance [21,22]. Controlling the membrane thickness and orientation is crucial for optimizing separation efficiency. A carefully adjusted membrane thickness can balance mechanical stability and permeability, ensuring that the membrane remains robust while still allowing efficient molecular transport [23,24,25]. Moreover, the orientation of MOF crystals within the membrane can be engineered to favor specific transport pathways, potentially increasing the selectivity and efficiency of the separation process [26,27,28]. Therefore, it is imperative to rationally tune the physical microstructure and chemical functionality of the UiO-66 membrane to improve its separation performance and broaden its application. However, several challenges must be addressed to achieve these goals.

In this review, the progress of UiO-66 membranes, emphasizing the structural tuning in terms of the chemistry, pore architecture, and membrane morphology will be comprehensively discussed. While there exist several comprehensive reviews addressing MOF membranes, few focus specifically on the structural modifications of UiO-66 membranes. The primary aim of this review is to enhance the understanding of UiO-66 membranes and to provide valuable insights for the design of high-performance membranes through effective structural optimization.

## 2. Synthesis of UiO-66 Crystal and UiO-66-Based Membranes

### 2.1. Synthesis of UiO-66 Crystal

Lillerud et al. first reported the synthesis of UiO-66 by mixing zirconium tetrachloride salt and H_2_BDC, followed by dissolution in N,N′-dimethylformamide (DMF) [9]. The resulting mixture was subsequently heated in a sealed container overnight. Upon completion of the crystallization, the solid product was filtered and thoroughly washed with DMF. As this marked the first generation of Zr-MOFs, there remained significant scope for exploration regarding the optimization of the synthesis recipe to achieve optimal properties, as well as to gain a deeper understanding of the underlying mechanisms involved in the synthesis process. Early attempts without the use of modulators or deprotonating agents resulted in rapid reactions, especially at higher concentrations, producing a gel-like product instead of the desired crystalline powder. This was due to rapid nucleation and the formation of a three-dimensional network with insufficient long-range order, leading to an amorphous structure.

A seminal work describing how modulators may be used as additives was presented by Schaate et al. in 2011, who demonstrated the use of modulators as additives to refine the crystallization process [29,30,31]. These modulators typically consist of a single carboxylic acid group attached to a carbon chain, with a general formula of R-COOH. The R group can vary widely, ranging from a methyl group or a hydrogen atom to more complex structures like a benzene ring or even a trifluoromethyl group. These modulators bind to the metal nodes, preventing the propagation of the crystal structure due to the absence of a second carboxylic acid group, thereby controlling the crystallization process and improving the overall quality of the MOF. Following this, Zhao et al. introduced deprotonating agents to further improve the synthesis [32]. Deprotonating agents play a crucial role in “activating” ligands and promoting nucleation during MOF synthesis, which is essential for controlling crystal growth. Triethylamine (TEA), commonly used for this purpose, acts as a base to remove a proton from the BDC ligand, thereby enhancing nucleation efficiency and resulting in more uniform and well-defined crystal growth. The combination of modulators and deprotonating agents has significantly advanced UiO-66 synthesis, enabling the production of high-quality crystals with desirable properties for various applications [33,34].

Years of research have allowed for facile tuning of UiO-66 crystal sizes, now ranging from as small as 10 nm to several tens of micrometers or larger [35,36,37]. Additionally, synthesis conditions have been greatly optimized. While early methods relied on prolonged solvothermal synthesis in DMF over hours or days, more recent approaches enabled rapid synthesis at room temperature in just a few minutes. These improved methods often utilize ethanol or even aqueous solutions, significantly enhancing the efficiency and scalability of UiO-66 crystal production.

### 2.2. Synthesis of Polycrystalline UiO-66 Membrane

The synthesis of UiO-66 membranes marked a breakthrough in 2015. Liu et al. successfully fabricated well-intergrown UiO-66 membranes on α-alumina hollow fibers through in-situ growth method [38]. Their work demonstrated that by optimizing preparation parameters such as the composition of the precursor solution, synthesis duration, and choice of substrates, high nucleation density and satisfactory intergrowth could be achieved. A pivotal factor in this synthesis was the presence of water in the mother solution, as the secondary building unit (SBU) of UiO-66 contains both OH^–^ and O^2–^ ions. Following the advancements in UiO-66 synthesis, several continuous UiO-66 membranes were successfully fabricated on a variety of substrates, significantly expanding their potential applications.

**Figure 3:**
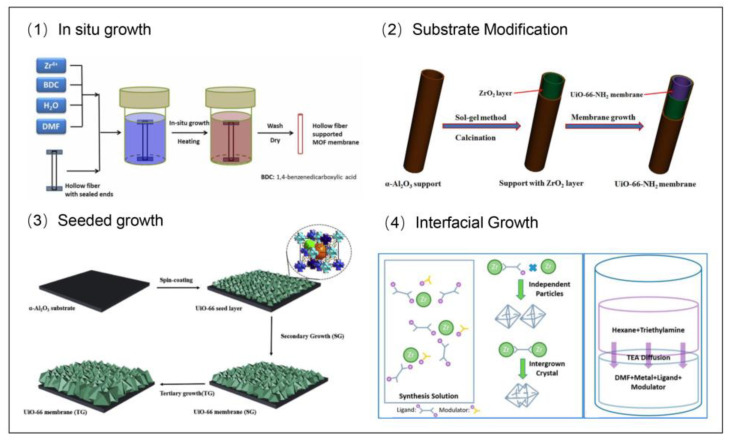
Typical strategies for the fabrication of UiO-66 membranes [38,41,42,43].

One of the primary challenges in synthesizing high-quality UiO-66 membranes lies in the precise control of heterogeneous nucleation, crystallization, and intergrowth on the substrate surface, while concurrently minimizing the formation of nonselective intercrystalline pinholes [39]. To address this issue, a range of modulated synthesis techniques has been developed, enabling tailored control over the structural integrity and performance of the membranes. These techniques involve the use of modulators such as formic acid, acetic acid, or benzoic acid, which serve to inhibit the coordination interactions between Zr⁴⁺ ions and BDC ligands [40]. These modulators effectively adjust the rates of nucleation and crystal growth, thereby enhancing the reproducibility of synthesis procedures and enabling the fine-tuning of key crystal characteristics, including size, morphology, and crystallinity. Additionally, typical methods for enhancing nucleation density and promoting crystal growth are detailed below (Figure 3), providing further insights into the optimization of UiO-66 membrane synthesis.

#### 2.2.1. Substrate Modification

Achieving a well-intergrown UiO-66 membrane structure through simple in-situ method approach can be difficult because of inadequate heterogeneous nucleation on the substrate [44]. To overcome this, substrate modifications such as grafting amine groups or precoating a zirconia (ZrO_2_) layer on the substrate can significantly enhance nucleation density and promote preferential crystallization on the substrate. For instance, Zhang et al. prepared UiO-66-NH_2_ membranes on α-alumina substrates modified with a thin layer of ZrO_2_, which favored heterogeneous nucleation [41]. The resulting membranes exhibited excellent performance in pervaporation desulfurization, with high flux and reproducibility for n-octane containing thiophene at 40 °C.

#### 2.2.2. Seeded Growth

Seeded growth is a commonly utilized technique for the synthesis of MOF membranes. This method involves applying a seeding layer onto the substrate prior to crystal growth, which serves to create enough nucleation sites. A uniform, high-density seeding layer that firmly adheres to the substrate is essential for the successful fabrication of MOF membranes. Various methods for fabricating the seeding layer have been explored, including dip coating, reactive seeding, air-liquid interface-assisted self-assembly, and sonication-driven seeding [45].

Additionally, techniques like microwave-assisted solvothermal growth and counter-diffusion-assisted epitaxial growth have been employed to facilitate crystal intergrowth. For instance, Liu et al. prepared UiO-66 membranes on α-alumina substrates precoated with UiO-66 seed layer. The resulting membranes exhibited excellent performance in CO_2_/N_2_ separation [42].

#### 2.2.3. Interfacial Growth

Another approach to control the nucleation and crystallization of UiO-66 on the substrate is by limiting the contact between the ligand and metal ion at a defined interface. Interfacial “polymerization” and contra-diffusion techniques have been proven effective for this purpose. For example, Mu et al. developed a biphase solvothermal reaction system with the addition of trimethylamine (TEA) as a deprotonating agent [43]. By designing a hexane-DMF biphase system where TEA was dissolved in the hexane phase and diffused into the DMF phase containing the metal and ligand sources, controlled deprotonation of the ligands was achieved. The facilitated crystal intergrowth, resulting in well-intergrown (200)-and (111)-oriented UiO-66 membranes with tunable properties.

## 3. Structural Manipulation of UiO-66 Membrane

To achieve high-efficiency separation, both the physical and chemical structures of UiO-66 membranes can be precisely engineered to optimize diffusion and adsorption processes. These manipulation strategies can be further divided into different groups: (1) modifications to the crystal lattice structure at the sub-nanometer scale, such as lattice flexibility, ligand structure, and membrane crystallinity, and (2) structure manipulation at the nanoscale, which include physical morphology and chemical properties, without disrupting the intrinsic structure of UiO-66. Advanced methods and advantages for structural manipulation of UiO-66 membranes are discussed below (Table 1).

### 3.1. Structure Influence on the Separation Performance

(1) Oriented polycrystalline MOF membranes are gaining popularity due to their ability to minimize grain boundary defects while enhancing gas separation performance. This improvement is achieved through the controlled tuning of crystallographic orientation in relation to the substrate [63]. The alignment of crystals within these membranes can be characterized by assessing different crystallographic preferred orientations (CPOs). This characterization involves a comparative analysis of the statistically oriented powder and the supported membrane layer, allowing for the determination of how well the crystals are aligned.

(2) One of the primary objectives in MOF polycrystalline membrane synthesis is to enhance their competitiveness for practical applications by achieving high permeance, reducing synthesis time, and facilitating scalable fabrication processes. A widely adopted strategy to improve permeance involves minimizing the thickness of defect-free membranes [23]. Notably, recent advancements have led to the development of ultrathin UiO-66 membranes with thicknesses reported to be less than 1 μm, and in some cases, even much thinner.

(3) Pore engineering plays a pivotal role in determining the performance of MOF membranes by enhancing their specific surface area, improving mass transfer efficiency, and modifying the interactions between the material and its surrounding environment [64]. These modifications can significantly influence the separation performance of the membranes. Through pore engineering-whether by manipulating pore size, functionalizing pore surfaces, or introducing hierarchical structures, UiO-66 membranes can be tailored to optimize separation performance for various molecular and ionic species.

(4) Defects in MOFs have been shown to significantly enhance performance across a diverse range of applications, including adsorption, catalysis, electronics, and magnetism [65]. These improvements can largely be attributed to increased porosity and the introduction of active sites. Consequently, defective MOFs offer a versatile platform for tuning their properties, going beyond mere compositional variations or structural perfection. In particular, defect engineering in MOFs, especially within membrane systems, has emerged as an efficient and effective strategy for improving separation performance.

### 3.2. Structural Manipulation Strategy

#### 3.2.1. Orientation and Thickness Engineering

**Figure 4:**
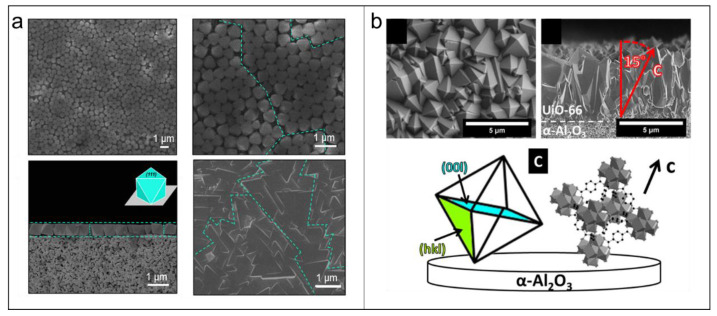
(**a**) SEM images of (111)-oriented UiO-66 seed layer and membrane [66]. (**b**) SEM images of UiO-66 membrane with a (002) preferred orientation [50].

Compared to in-situ methods, epitaxial growth offers precise control over the preferred orientation of MOF membranes by combining the pre-deposition of a MOF seed layer with controlled epitaxial growth. This approach allows for two common growth mechanisms: oriented epitaxial growth and evolutionary growth. The former involves epitaxial growth that follows the crystallographic orientation inherited from the seed layer. The latter involves van der Drift evolutionary selection originating from randomly oriented seed layer, during which the fastest growth direction determines dominant out-plane membrane orientation through competitive epitaxial growth. For example, Liu et al. developed a dynamic air-liquid interface-assisted self-assembly method to deposit highly oriented UiO-66 seed layers on porous α-Al_2_O_3_ substrates (Figure 4). By combining controlled epitaxial growth with a novel zirconium source (e.g., ZrS_2_, Zr clusters), they successfully prepared a highly (111)-oriented UiO-66 membrane [66,67]. In contrast, Caro et al. synthesized a UiO-66 membrane with a (002) preferred orientation by using a randomly oriented seed layer based on evolutionary growth [50].

**Figure 5:**
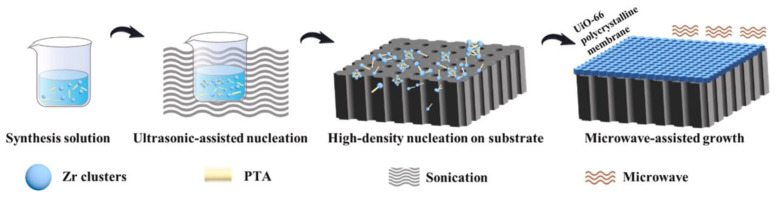
Schematic diagram of the UiO-66 polycrystal membrane synthesis with ultrasonic-assisted nucleation followed by microwave-assisted membrane growth [48].

Wei et al. demonstrated a novel synthesis approach for ultrathin UiO-66 membranes, achieving an impressive thickness of only 210 nm. The method leveraged sonication to generate a high density of nucleation sites, which is subsequently complemented by rapid membrane growth through microwave-assisted techniques [48,68]. The elevated temperatures near the substrate help to drive the nucleation and growth of the MOF crystals, which is essential for forming a uniform and defect-free membrane (Figure 5). The resulting UiO-66 membrane exhibited excellent performance, maintaining a Na^+^ rejection rate of 99.6% for 700 h during a long-term experiment, with a water flux of 0.16 L/(m^2^·h·bar) in forward osmosis.

**Figure 6:**
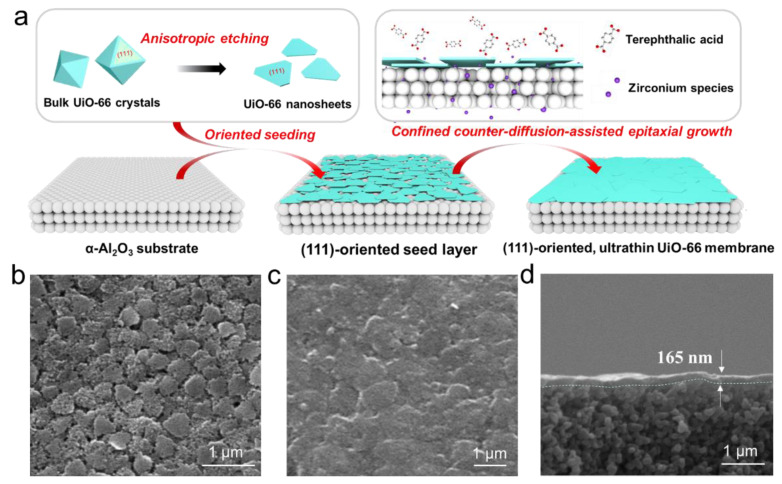
(**a**) The procedure for the preparation of highly (111)-oriented ultrathin UiO-66 membrane. (**b**) SEM image of UiO-66 nanosheet seed layer and (**c**,**d**) UiO-66 membrane prepared by confined counter-diffusion epitaxial growth [24].

Recently, Liu et al. introduced an innovative approach to fabricate uniform triangular-shaped 40 nm-thick UiO-66 nanosheet seeds using an anisotropic etching strategy (Figure 6). They further developed a confined counter-diffusion-assisted epitaxial growth method to create a highly (111)-oriented UiO-66 membrane with a thickness of just 165 nm [24]. The significant reduction in thickness and diffusion barriers endowed the membrane with unprecedented CO_2_ permeance (2070 GPU) and high CO_2_/N_2_ selectivity (35.4), surpassing the performance limits of current polycrystalline MOF membranes. However, fabricating UiO-66 membranes with a thickness of less than 100 nm remains a significant challenge, requiring further innovation and optimization in synthesis techniques.

#### 3.2.2. Pore Engineering

(1) Rational Design of Pore Size

**Figure 7:**
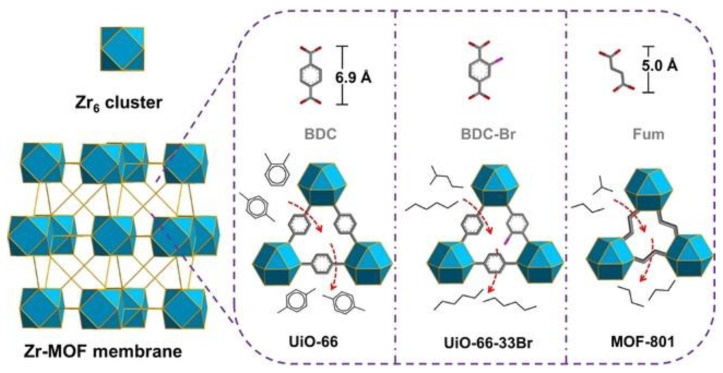
Scheme of UiO-66 membranes with different functional group for isomer separation [60].

One effective approach to pore engineering in MOF membranes involves the incorporation of functional groups that exert steric effects on the pore structure. For instance, Sivaniah et al. employed coordination modulation techniques to synthesize UiO-66 membranes characterized by diverse ligand chemistry and functionality [59]. By incorporating bulkier organic ligands, such as H_2_NDC and H_2_ADC, the selectivity for hydrogen (H_2_) was improved through the molecular sieving effect. Molecular simulations demonstrated that the introduction of additional benzene rings in the MOF structure resulted in constricted pore apertures, which effectively reduced the diffusivity of larger molecules while maintaining minimal impact on hydrogen transport, resulting in an improved gas mixture separation factor of H_2_/CO_2_ = 26. Liu et al. employed rational design to fine-tune the pore size of Zr-MOF membranes, specifically targeting the separation of isomers [60]. To enhance the separation of n-hexane from 2-methylpentane, the pore size of UiO-66 membranes was strategically reduced through adjustments in the functional groups and ligand proportions, leading to the development of UiO-66-33Br membranes (Figure 7). The resulting membranes demonstrated optimized pore sizes that exhibited an exceptional n-hexane/2-methylpentane selectivity of 9.10, coupled with a n-hexane permeance of 49.1 GPU. Recently, Xu et al. synthesized a UiO-66 membrane incorporating dibenzo-18-crown-6 (DB18C6) within its cavity, creating a system that integrated size sieving and interaction screening [61]. This novel approach significantly enhanced ion permeability while maintaining high selectivity. Notably, the DB18C6@UiO-66 membrane demonstrates K⁺ permeation rate of 1.2 mol m^−2^ h^−1^ and K⁺/Mg^2^⁺ selectivity ratio of 57.

(2) Modifying Pore Surface Chemistry

In addition to adjusting pore size, modifying the inner surfaces of the pores can improve host-guest interactions, enabling selective separation of specific components. Li et al. reported high-precision separation of monovalent and divalent cations using functionalized MOF membranes, specifically UiO-66-(X)_2_, where X = NH_2_, SH, OH, or OCH_3_. The functional groups and the sub-nanochannel sizes synergistically regulate ion binding affinity and dehydration processes, which significantly enhance selectivity. Notably, the UiO-66-(OCH_3_)_2_ membrane achieved a remarkable K⁺/Mg^2^⁺ selectivity of 1567.8 [21].

(3) Introducing Hierarchical Structures

**Figure 8:**
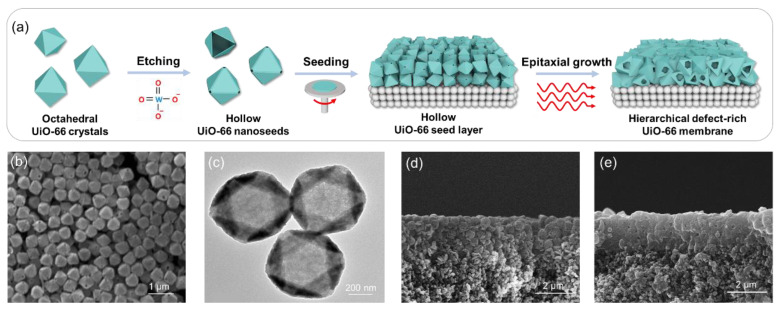
(**a**) Schematic illustration of the fabrication of hierarchical UiO-66 membranes through epitaxial growth. (**b**) SEM and (**c**) TEM image of hollow UiO-66 seeds. (**d**) SEM images of hollow UiO-66 seed layer. (**e**) SEM images of hierarchical UiO-66 membrane prepared by single-mode microwave heating at 100 °C for 30 min [57].

Another promising strategy for pore engineering involves the introduction of mesopores within the UiO-66 membrane to reduce mass transfer resistance and improve separation efficiency. This hierarchical structure helps alleviate diffusion resistance by shortening the diffusion path length for guest molecules. Recently, Liu et al. developed a hierarchical UiO-66 membrane by synergistically combining hollow-structured UiO-66 seeds with microwave-assisted epitaxial growth methods [57]. The resulting membrane exhibited a (111)-oriented top selective layer, which was supported by a lower hollow layer on a porous α-Al_2_O_3_ substrate (Figure 8). This design reduced the diffusion path length while increasing missing-linker defects, resulting in a CO_2_/N_2_ selectivity of 38.1 and a CO_2_ permeance of 2170 GPU.

#### 3.2.3. Defects Engineering

Liu et al. recently prepared defect-rich (111)-oriented UiO-66 membrane by combining defect engineering with tertiary growth approach. The utilization of ZrS_2_ as the metal source during solvothermal synthesis led to an increase in missing-linker defects within the MOF framework [69]. These defects contributed to an elevated BET area and pore volume, alongside the creation of additional active sites. These features were critical for enhancing CO_2_ adsorption. Following the tertiary growth process, the defective UiO-66 membrane demonstrated CO_2_ permeance of 4.07 × 10^−7^ mol m^−2^ s^−1^ Pa^−1^ and exhibited CO_2_/N_2_ selectivity of 35.6. However, the synthesis of defective UiO-66 membranes is still limited by the necessity for high-temperature conditions. To address this limitation, Liu et al. subsequently developed a room-temperature synthesis strategy that employed pre-synthesized Zr_6_O_4_(OH)_4_ clusters as zirconium source. This innovative approach effectively reduces the activation energy required for the synthesis process, thereby allowing for more precise control over the number of missing-linker defects [52]. The defect density of the resulting UiO-66 membrane was tunable by adjusting the reaction temperature or the ratio of organic linkers, resulting in CO_2_/N_2_ selectivity of 37.8, along with a CO_2_ permeance of 2.11 × 10^−8^ mol m^−2^ s^−1^ Pa^−1^.

**Figure 9:**
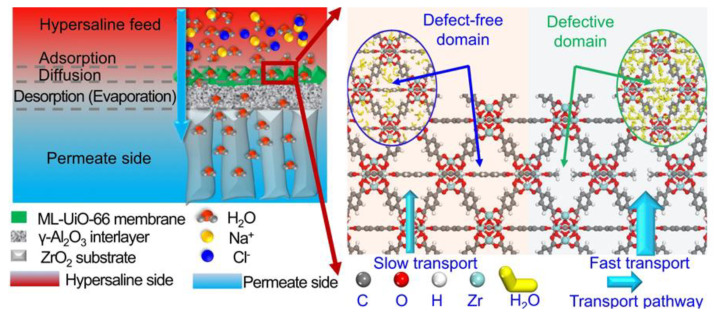
(**a**) Pervaporation desalination process in ultrathin UiO-66 membrane and (**b**) mechanism of intra-crystalline defect-enhanced water permeation [53].

Further developments by Dong et al. explored the fabrication of ultrathin UiO-66 membranes with missing-linker defects by utilizing CH_3_COOH as a growth modulator [53]. Their findings confirmed that the monocarboxylate group compensates for missing-linker defects, resulting in an increase in specific surface area from 990.4 to 1249.0 m^2^ g^−1^ and a corresponding enlargement in pore size from 0.508 to 0.568 nm. The structural hydrophilicity of the sub-nanometer channels was also improved, facilitating rapid water transport (Figure 9). The resulting ultrathin membranes exhibited nearly complete salt rejection, with water fluxes reaching 29.8 L m^−2^ h^−1^, outperforming other leading zeolite and MOF membranes.

**Figure 10:**
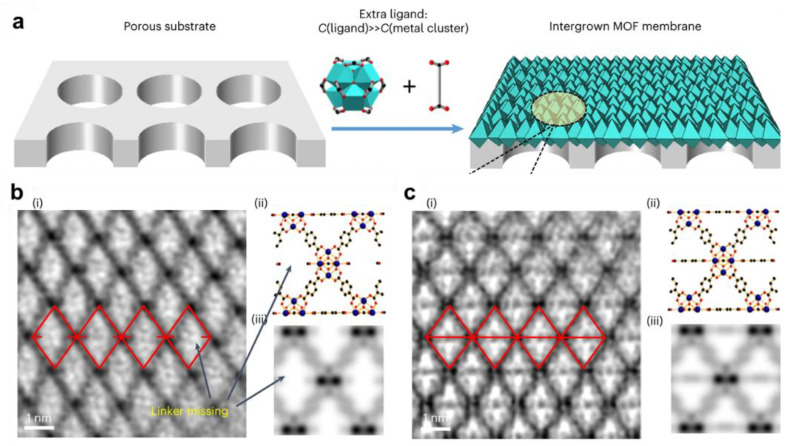
(**a**) Schematic illustration of the fabrication of UiO-66 membranes with perfect lattices. (**b**) High-resolution TEM images of UiO-66 along the [110] zone axes with a defective lattice and (**c**) a perfect lattice. (**i**) Contrast transfer function-corrected image. (**ii**) Projected structural model. (**iii**) Simulated projected potential [50].

In contrast, Jin et al. pursued a defect-elimination approach aimed at enhancing size-sieving separation in MOF membranes [54]. Their method employed a theoretical coordination strategy to overcome steric hindrances associated with fully connecting ligands to metal clusters (Figure 10). By systematically varying the stoichiometric ratios of ligands to SBUs between 1.5 and 20, they achieved water/salt selectivity as high as 9000, with water fluxes of approximately 10 L m^−2^ h^−1^. Overall, defect engineering in MOF membranes presents a dichotomy: defects can increase permeance, while the elimination of defects can enhance size sieving selectivity. This trade-off must be carefully managed, particularly for applications such as desalination and gas separation, where both high permeance and selectivity are critical. The optimization of these parameters will be key to advancing the practical implementation of MOF-based membranes in industrial settings. Recently, Zhao et al. utilized a bimetallic method to prepare a biUiO-66 membrane featuring distinct reo-topology frameworks characterized by periodic missing-cluster defects [55]. The regulation of the reo structure was achieved by adjusting the synthesis temperature and the Zn/Zr molar ratio. Molecular-sieving experiments demonstrated that the introduction of missing-cluster defects facilitates the precise discrimination of complex mixtures with molecular weights below 350 g mol^−1^.

### 3.3. Structural Design of UiO-66 Membranes Towards Different Applications

The effective aperture size and the nature of functional groups within UiO-66 type MOFs play a crucial role in determining the membrane separation capability, as predicted by the molecular sieving and adsorption-diffusion mechanism. In this context, we discuss three primary categories of applications based on membrane processes: gas separation, water treatment, and ion separation. Each of these applications benefits from the unique structural characteristics and functionalization of UiO-66, highlighting its potential for advancements in separation technology.

#### 3.3.1. Gas Separation

UiO-66 membranes have shown considerable promise in the field of gas separation due to their unique structural characteristics and tunable properties. The potential of these membranes for separating gas mixtures can be attributed to various mechanisms, including molecular sieving, selective adsorption, and facilitated transport. Therefore, regulating the pore structure, with an emphasis on both the pore size and the functional pore surface, emerges as an effective strategy for enhancing separation selectivity. Based on this pore engineering approach, UiO-66 membranes have been developed for the separation of CO_2_/N_2_ [24], H_2_/CO_2_ [61], and hydrocarbon isomers [60].

#### 3.3.2. Water Treatment

Given its excellent humidity stability and molecule-sized pores, UiO-66-based membranes have found extensive applications in water treatment. Specifically, they are capable of handling salt-aqueous solutions (such as in desalination processes), dye (polar compound)-laden solutions (for the purpose of dye removal), and protein-aqueous solutions (in the context of protein removal). On one hand, by incorporating hydrophilic functional sites into the framework, the water affinity is significantly enhanced. This, in turn, leads to a notable increase in permeability [53]. On the other hand, the pore sizes of UiO-66 membranes can be meticulously designed. This allows for the selective transport of water molecules while effectively excluding larger salt ions, dyes, or proteins [70]. At the same time, the interaction between the charged salt ions or dye molecules and the functionalized UiO-66 framework results in significant selectivity advantages. As a result, the rejection efficiencies are further enhanced when compared to conventional membrane materials.

#### 3.3.3. Ion Separation

The pore size of UiO-66 can be precisely customized through the selection of different organic linkers or by adopting a mixed-linker approach. This enables the design of pores that are precisely tailored to the size of the target metal ions [58]. Specifically, a pore size within the range of 6–12 Å is typically effective in selectively permitting the passage of smaller hydrated metal ions while effectively excluding larger contaminants. Moreover, by employing post-synthetic modification techniques, such as covalent grafting or ion exchange, functional groups can be introduced [18,59]. These functional groups enhance the interactions with specific metal ions. For instance, the introduction of amino groups can significantly increase the framework’s affinity for cationic metal ions [71], thereby promoting higher separation efficiencies. This approach provides a powerful means to fine-tune the pore properties of UiO-66 and optimize its performance in metal ion separation processes.

## 4. Summary and Outlook

This review highlights the significant strides made in the engineering and design of UiO-66 membranes for advanced separation applications. UiO-66, with its exceptional stability, high surface area, and tunable pore structures, has emerged as a leading material for membrane applications. Key advancements include refined synthesis methods, such as the use of modulators and deprotonating agents to improve crystal quality and membrane performance. Structural manipulations, including orientation control, pore engineering, and defect engineering have further enhanced the separation efficiency of UiO-66 membranes for gas and liquid applications.

Despite these advancements, several challenges remain:

First, further increasing the orientation of UiO-66 membranes. Although several strategies have been developed to achieve this destination, the current protocol still suffers from accurate seed morphology and epitaxial growth kinetics control, limiting the available types of oriented UiO-66 membranes.

Second, facile and cost-effective preparation of UiO-66 membranes on a large scale. To achieve precise control over membrane structure, most of the current protocols remain comparatively complicated, resulting in lower reproducibility, and therefore, higher production cost.

Third, optimizing the balance between defect density and membrane selectivity continues to be an area of active research. Future efforts should focus on addressing these challenges through innovative synthesis techniques, improved understanding of defect-engineering impacts, and scaling up fabrication processes for industrial applications.

Finally, several key conclusions should be considered for future research toward oriented MOF-based membranes. (i) Future research is anticipated to focus on the development of MOFs with adjustable pore sizes and functional groups, optimizing them for specific applications such as gas separation, water treatment, and ion removal. (ii) Developing cost-effective and efficient synthesis methods will be crucial for transitioning from laboratory-scale studies to practical applications. (iii) The future of MOFs in membrane technology may also involve their integration with advanced processes such as membrane distillation, pervaporation, and nanofiltration. Coupling MOFs with nanotechnology could lead to the development of mem-branes exhibiting enhanced separation capabilities and improved energy efficiency.

## Figures and Tables

**Table 1:** Summary of UiO-66 membranes in terms of structure character, synthetic approaches, applications, and separation performance.

Materials	Structure Character	Synthetic Approaches	Application	Separation Performance	Ref.
UiO-66	2.0 μm thick Randomly oriented	In situ synthesis	Nanofiltration (K^+^, Na^+^, Ca^2+^, Mg^2+^, Al^3+^)	Mg^2+^ rejection = 98.0%, Al^3+^ rejection = 99.3% Water permeance: 0.14 L m^−2^ h^−1^ bar^−1^	[38]
UiO-66-NH_2_	4 µm thick Randomly oriented	In situ synthesis (modified substrates)	Pervaporation (thiophene, n-octane)	n-octane flux: 2.16 kg m^−2^ h^−1^ thiophene/n-octane = 17.86	[42]
UiO-66-NH_2_	4–6 μm thick Randomly oriented	Contra diffusion	Electrodialysis (Cl^−^, SO_4_^2−^)	Cl^−^/SO_4_^2−^ = 36.23	[46]
UiO-66	400 nm thick	Secondary growth	Pervaporation (MeOH, MTBE)	MeOH flux: 5.68 kg m^−2^ h^−1^ MeOH/MTBE = 28,000	[47]
UiO-66	210 nm thick	Microwave-assisted secondary growth	Ions rejection (Li^+^, Na^+^, Ca^2+^, Mg^2+^, Al^3+^)	Na^+^ rejection = 99.6% water flux: 0.16 L m^−2^ h^−1^ bar^−1^	[48]
UiO-66-SO_3_H	600 nm thick	in situ growth	Electrodialysis (K^+^, Na^+^, Mg^2+^)	Na^+^/Mg^2+^ = 170	[49]
UiO-66	(111)-Orientated 165 nm thick	Confined contra-diffusion -assisted epitaxial growth	Gas separation (H_2_, CO_2_, N_2_, CH_4_)	CO_2_ permeance: 2070 GPU CO_2_/N_2_ = 35.4	[24]
UiO-66	(002)-Orientated 2.0 μm thick	Secondary growth	Gas separation (H_2_, CO_2_, N_2_, CH_4_, C_2_H_6_, C_3_H_8_)	H_2_/CO_2_ = 5.1, H_2_/N_2_ = 4.7, H_2_/CH_4_ = 12.9, H_2_/C_2_H_6_ = 22.4, H_2_/C_3_H_8_ = 28.5	[50]
UiO-66	300 nm thick	Cathodic deposition	Electro-chemical ion separation (Li^+^, K^+^, Na^+^, Ca^2+^, Mg^2+^)	Li^+^/Mg^2+^= 286 Li^+^ permeance: 11.2 _mol_ m^−2^ h^−1^	[51]
UiO-66	Introducing missing-cluster defects	Secondary growth	Gas separation (H_2_, CO_2_, N_2_, CH_4_)	CO_2_/N_2_ = 37.8, CO_2_ permeance: 2.11 × 10^−8^ mol m^−2^ s^−1^ Pa^−1^	[52]
UiO-66	Introducing missing-cluster defects	In situ growth	Desalination (Na^+^, Cl^−^)	NaCl rejection > 99.8% water flux: 29.8 L m^−2^ h^−1^	[53]
UiO-66	Eliminating lattice defects	Contra diffusion	Desalination/pervaporation (K^+^, Na^+^, Ca^2+^, Mg^2+^) (MeOH, DMC)	water/salt selectivity 9000, water flux: 10 L m^−2^ h^−1^ DMC rejection > 99.5% MeOH flux: 17.71 kg m^−2^ h^−1^	[54]
reo-UiO-66	Introducing missing-cluster defects	In situ growth	Pervaporation (MeOH, DMC)	MeOH permeance: 11.2 L m^−2^ h^−1^ bar^−1^ EB rejection > 97.8%	[55]
UiO-66	Healing lattice defects	Tertiary growth	Pervaporation (MeOH, MTBE, EtOH, ETBE)	MeOH/MTBE = 10,000 MeOH flux: 2.14 kg m^−2^ h^−1^	[56]
UiO-66	Hierarchical defect-rich pore	Microwave-assisted secondary growth	Gas separation (H_2_, CO_2_, N_2_, CH_4_)	CO_2_/N_2_ = 38.1 CO_2_ permeance: 2170 GPU	[57]
UiO-66@NTDS	Molecules incorporated pore	In-situ growth	Ion separation (Li^+^, K^+^, Na^+^, Mg^2+^)	K^+^/Mg^2+^ = 73 Na^+^/Mg^2+^ = 57 Li^+^/Mg^2+^ = 46	[58]
UiO-66-(OCH_3_)_2_	Molecularly tailored functional group and pore size	In-situ growth	Electro-chemical ion separation (Li^+^, K^+^, Na^+^, Ca^2+^, Mg^2+^)	K^+^/Mg^2+^ = 1657.8 K^+^ permeance: 0.05 mol m^−2^ h^−1^	[21]
UiO-66	Bulkier organic ligands incorporated pore (NDC, ADC)	In-situ growth	Gas separation (H_2_, CO_2_, N_2_, CH_4_)	H_2_/CO_2_ = 26	[59]
UiO-66-33Br	Molecularly tailored functional group and pore size	In-situ growth	Gas separation (n-hexane, 2-methylpentane)	n-hexane/2-methylpentane = 9.10 n-hexane permeance: 49.1 GPU	[60]
DB18C6@UiO-66	Crown ether confined pore	In-situ growth	Ion separation (Li^+^, K^+^, Na^+^, Mg^2+^)	K^+^/Mg^2+^ = 57 K^+^ permeance: 1.2 mol m^−2^ h^−1^	[61]
UiO-66-NH_2_-SA	Pore modification with salicylaldehyde (SA)	Secondary growth	Pervaporation (MeOH, Tol, MTBE)	MeOH/Tol = 3220 MeOH/MTBE = 28,000	[62]

## Data Availability

The raw data supporting the conclusions of this article will be made available by the authors on request.
